# Neuronal expression of pathological tau accelerates oligodendrocyte progenitor cell differentiation

**DOI:** 10.1002/glia.22940

**Published:** 2015-11-18

**Authors:** Bernardino Ossola, Chao Zhao, Alastair Compston, Stefano Pluchino, Robin J. M. Franklin, Maria Grazia Spillantini

**Affiliations:** ^1^ Department of Clinical Neurosciences, Clifford Allbutt BuildingUniversity of CambridgeCambridge CB2 0AHUnited Kingdom; ^2^ Wellcome Trust‐Medical Research Council Cambridge Stem Cell Institute, University of CambridgeCambridgeCB2 0AHUnited Kingdom; ^3^ NIHR Biomedical Research CentreCambridgeCB2 0AHUnited Kingdom

**Keywords:** OPCs axons communication, axonal damage, remyelination, tau pathology, inflammation, microgliosis, OPCs priming, regenerative environment

## Abstract

Oligodendrocyte progenitor cell (OPC) differentiation is an important therapeutic target to promote remyelination in multiple sclerosis (MS). We previously reported hyperphosphorylated and aggregated microtubule‐associated protein tau in MS lesions, suggesting its involvement in axonal degeneration. However, the influence of pathological tau‐induced axonal damage on the potential for remyelination is unknown. Therefore, we investigated OPC differentiation in human P301S tau (P301S‐htau) transgenic mice, both *in vitro* and *in vivo* following focal demyelination. In 2‐month‐old P301S‐htau mice, which show hyperphosphorylated tau in neurons, we found atrophic axons in the spinal cord in the absence of prominent axonal degeneration. These signs of early axonal damage were associated with microgliosis and an upregulation of IL‐1β and TNFα. Following *in vivo* focal white matter demyelination we found that OPCs differentiated more efficiently in P301S‐htau mice than wild type (Wt) mice. We also found an increased level of myelin basic protein within the lesions, which however did not translate into increased remyelination due to higher susceptibility of P301S‐htau axons to demyelination‐induced degeneration compared to Wt axons. *In vitro* experiments confirmed higher differentiation capacity of OPCs from P301S‐htau mice compared with Wt mice‐derived OPCs. Because the OPCs from P301S‐htau mice do not ectopically express the transgene, and when isolated from newborn mice behave like Wt mice‐derived OPCs, we infer that their enhanced differentiation capacity must have been acquired through microenvironmental priming. Our data suggest the intriguing concept that damaged axons may signal to OPCs and promote their differentiation in the attempt at rescue by remyelination. GLIA 2016;64:457–471

## Introduction

Remyelination is the most efficient regenerative process of the adult CNS and is vital for functional recovery in demyelinating diseases like multiple sclerosis (MS) (Franklin et al., 2012). Following demyelination oligodendrocyte progenitor cells (OPCs), which are abundant in the adult CNS, migrate toward the lesion where they proliferate and differentiate into myelinating oligodendrocytes (Crawford et al., 2013). Remyelination becomes less efficient in the later stage of MS in part due to a failure of OPC differentiation (Franklin and ffrench‐Constant, 2008; Kuhlmann et al., 2008; Wolswijk, 1998). Failure of remyelination is a major contributor to the accumulation of axonal and neuronal degeneration that characterizes the progressive stage of the disease in which clinical deficits accumulate over time (Bjartmar et al., 2000; De Stefano et al., 2001). The axonal degeneration observed after toxin‐induced demyelination in mice depleted of OPCs is prevented by the transplantation of exogenous OPCs that restore remyelination capacity (Irvine and Blakemore, 2008). Thus, enhancing remyelination, and particularly OPC differentiation, is an important therapeutic strategy to promote neuroprotection in the progressive stage of MS. Injured axons in the MS lesion initially show a focal mitochondrial dysmorphism and axonal swelling (Nikic et al., 2011) which subsequently develops into axonal truncation with terminal ovoids (Trapp et al., 1998). Injured axons in MS lesions accumulate amyloid precursor protein (APP), which is detectable only in axons with impaired fast axonal transport (Ferguson et al., 1997; Sherriff et al., 1994). The impairment of axonal transport may result from the recently demonstrated presence in MS lesions of hyperphosphorylated tau (Anderson et al., 2008).

Tau is a microtubule‐associated protein that when abnormally hyperphosphorylated loses its function of promoting microtubule stabilization altering axonal transport as observed in primary cultures (Bramblett et al., 1993; Mellone et al., 2013), in human IPSC‐derived neurons (Iovino et al., 2015), and in P301S‐htau mice (Bull et al., 2012). The presence of hyperphosphorylated filamentous tau is a feature of several neurodegenerative disorders such as Alzheimer's disease, frontotemporal dementia and parkinsonism linked to chromosome 17 (FTDP‐17T), progressive sopranuclear palsy, and others that are collectively designated as tauopathies (Spillantini and Goedert, 2013). The dysfunction of tau is sufficient to cause neurodegeneration, as shown in FTDP‐17T that is caused by mutations in the *MAPT* gene (Spillantini et al., 1998; Hutton et al., 1998).

The inflammatory attacks that repeatedly occur in MS are likely to result in tau post‐translational alterations, since an inflammatory environment is clearly associated with tau hyperphosphorylation in animal studies (Bellucci et al., 2004; Bhaskar et al., 2010; Birch et al., 2014). In addition hyperphosphorylated insoluble tau has been observed in several cases of primary and secondary progressive MS but not in an example of relapsing remitting MS (Anderson et al., 2008, 2009, 2010). In a mouse model of chronic inflammation‐driven demyelination the levels of insoluble tau positively correlate with axonal loss (Anderson et al., 2008), suggesting that the conversion of soluble to insoluble tau may contribute to axonal pathology in the progressive stage of MS. The phosphorylation status of tau is finely regulated by several kinases and phosphatases (Hanger et al., 2009), and its hyperphosphorylation can be also physiological, for example during fetal brain development, hypothermia, and hibernation (Spillantini and Goedert, 2013). Thus, the presence of hyperphosphorylated tau can be reversible and is not necessarily a precursor to insoluble aggregated forms. Reversible injury may also be a sign of early axonal degeneration in demyelinated lesions, as demonstrated in a mouse model of inflammation‐driven demyelination where some axons with focal swelling and dysmorphic mitochondrial recover spontaneously (Nikic et al., 2011). Thus, myelin can rescue damaged axons by stabilising the cytoskeleton, releasing trophic factors, and providing a source of energy such as lactate (Franklin et al., 2012; Lee et al., 2012). Conversely, axons also signal to oligodendrocytes and OPCs, for example by releasing glutamate that in the presence of the growth factor neuregulin promotes myelination and remyelination (Demerens et al., 1996; Lundgaard et al., 2013). However, whether the signals from damaged axons may also affect remyelination remains unclear.

In this study we investigated whether axonal damage associated with tau pathology affects remyelination. In particular we investigated OPC differentiation and remyelination following a toxin‐induced focal demyelination in P301S‐htau mice that express the human tau transgene under the pan‐neuronal promoter Thy1.2 (Allen et al., 2002). We also confirmed the behavior of OPCs isolated from P301S‐htau mice *in vitro*.

## Materials and Methods

### Antibodies

Primary antibodies: HT7 (mouse, Thermo Scientific, 1:1,000), AT8 (mouse, Thermo Scientific, 1:1,000), NF200 (rabbit, Sigma–Aldrich, 1:1,000), APP (clone 22C11, mouse, Millipore, 1:1,000), Iba1 (rabbit, Wako, 1:5,000), CD11b (rat, Serotec, 1:1,000), Olig2 (rabbit, Millipore, 1:1,000), NG2 (rabbit, Millipore, 1:1,000) APC (mouse, clone CC1, Chalbiochem, 1:200), SOX2 (goat, Santa Cruz, 1:300), MBP (rat, Serotec, 1:300 in IHC, 1:500 for ICC, and 1:2,000 for WB), βIII‐tubulin (Rabbit, AbCam, 1:500), β‐actin (mouse, Sigma–Aldrich, 1:10,000). Biotin‐conjugated secondary antibodies (Vector, 1:1,000), Alexa‐conjugated secondary antibodies (Molecular Probes, 1:1000), HRP‐conjugated secondary antibodies (1:5000): anti‐mouse and anti‐rabbit (GE), anti‐rat (Vector).

### Animals

Control Wt (C57Bl/6S OlaHsd; Harlan) and homozygote P301S‐htau transgenic mice (kindly provided by Dr Michel Goedert, MRC Laboratory of Molecular Biology, Cambridge, UK) expressing under the Thy1.2 promoter the shortest human tau isoform with four repeats no N‐terminal inserts and with the P301S mutation (Allen et al., 2002), were kept in a 12 h light/dark cycle with water and food *ad libitum* (R105‐25, Safe). In each experiment we used the same ratio of female and male animals for Wt and P301S‐htau mice. All procedures were performed in accordance with the UK Animal (Scientific Procedures) Act 1986 for the welfare of laboratory animals and were approved by the Cambridge University local ethical committee.

### Focal Demyelination

Under anaesthesia with isoflurane 7‐ to 8‐week‐old mice received 1 μL of 1% lysolecithin (l‐*a*‐lysophosphatidylcholine) (Sigma–Aldrich) dissolved in sterile PBS into the ventral funiculus of the spinal cords at the level of T12‐13 (Zhao et al., 2006). The injection was performed with a 10 μL Hamilton syringe, fitted with a pulled glass capillary, at a rate of ∼1 μL min^−1^. For pain relief mice received an injection of buprenorphine (0.02 mg kg^−1^, s.c.) at the beginning of the procedure.

### Oligodendrocyte Precursor Cell (OPC) Cultures

OPCs were isolated from either 1 or 10‐ to 12‐day‐old mice (male and females) using A2B5‐conjugated microbeads positive sorting as previously described with some modifications (Windrem et al., 2004) (see Supporting Information). This isolation protocol yielded highly enriched OPC cultures with almost undetectable microglial and neuronal cells and with 4.8% ± 0.72% of astrocytes found after 4 days of proliferation.

#### Migration assay

Directly after isolation OPCs were placed on the top chamber of transwell (8 μm pores, Millopore, UK) in growth factor‐free OPC medium, while the bottom chamber was filled with OPC medium supplemented with twofold concentration of growth factor cocktail. After 12 h incubation at 37°C, we quantified the number of Hoechst 33258 (1 μg mL^−1^, Sigma–Aldrich) positive cells that crossed the transwell mash.

#### Proliferation assay

After 36 h of seeding, OPCs were incubated with the thymidine analogue 5‐ethynyl‐2'‐deoxyuridine (EdU, 10 μ*M* final concentration, Invitrogen) for 3 h at 37°C and subsequently fixed and stained with the Click‐iT kit (Invitrogen) conjugated with Alexa 488.

#### Cell death and differentiation assay

When reaching 50% confluence, 4–5 days after seeding, OPCs were incubated with growth factor‐free OPC medium which was replaced with fresh growth factor‐free OPC medium after 2 days. After two more days cells were fixed and stained for either DNA fragmentation (TUNEL technology, *In Situ* Cells Death detection kit, Roche) to assess cell death or, for MBP and Olig2, to quantify the number of mature oligodendrocytes.

### Immunohistochemistry and Immunocytochemistry

Tissues and cell cultures were prepared for immunostaining as previously described with some modifications (Brelstaff et al., 2015) (see Supporting Information).

#### APC immunohistochemistry

Sections on slides were incubated for 30 min at RT with antigen retrieval reagent (Sigma–Aldrich). Following cell permeabilization with 1% Triton X‐100 in TBST (TBS with 0.05% Tween‐20), tissue nonspecific staining was blocked for 30 min with 10% normal donkey serum (NDS) in a 0.25% Triton X‐100‐TBST. Nonspecific background was blocked with IgG solution from the MOM kit (Vector) 1:25 in TBST for 1 h at RT. Sections were then first incubated with APC (CC1 clone, 1:200 in TBST with 5% NDS) for 1 h at RT, followed by the exposure to the secondary antibody (Donkey anti‐mouse (anti‐IgGb), Alexa 488, 1:1,000) for 1 h at RT. Tissue was then treated as previously described above.

### *In Situ* Hybridization

The proteolipid protein (PLP) probe was created for *in situ* hybridization procedure as previously described (Sim et al., 2002). Cryostat sections of 12 μm thickness mounted on poly‐l‐lysine coated glass slides were incubated with the riboprobes overnight at 58°C. The excess unbound labelled probes were removed by washing three times at 65° C with 1× Saline‐sodium citrate (150 mM NaCl, 15 mM Sodium citrate, pH 7) supplemented with 50% formamide and 0.1% Tween 20 solution. Sections were further washed twice with maleic buffer (50 mM maleic acid, pH 7.5, 250 mM NaCl and 0.1% Tween 20), then incubated with a solution of blocking reagent (2%, Roche) and 20% sheep serum made in maleic buffer at RT, followed by overnight incubation in alkaline phosphatase conjugated antibody at 4°C in a humidified chamber. After washing with maleic buffer five times for 10 min, the signal was visualised by incubating the slides with staining buffer containing NBT/BCIP (Roche, Lewes, UK) using a standard protocol.

### Toluidine Blue Staining, Ranking Analysis, and Oil Red O Staining

Tissue blocks of spinal cord of mice perfused with 4% glutaraldehyde diluted in PBS were processed through osmium tetroxide, dehydrated, and embedded in TAAB resin (TAAB Laboratories, Aldermaston, UK). Alkaline toluidine blue dye (0.5% Toluidine Blue+ 0.5% Borax) (Sigma–Aldrich) was applied on semi‐thin (1 μm) sections and heated with a Bunsen flame for 10 sec before washing with tap water (Zhao et al., 2006).

Ranking analysis was performed on toluidine blue stained sections containing similar sized lesions by a researcher blinded to the sample identity as described previously (Ibanez et al., 2004). In brief, sections from different mice were compared by ranking with the higher score to the lesion that contained less demyelinated axons. Thus, this method refrained from assigning a value representing the proportion of remyelinated axons. The same rank was assigned to sections that contained similar amounts of demyelinated axons.

Oil red O stain kit (MasterTech) was used on 12 μm PFA‐fixed tissue sections prepared as described above.

### Western Blotting

Cells from OPCs cultures were incubated with RIPA buffer (25 mM tris pH 7.4, 150 mM NaCl, 1% Triton‐x 100, 1% Na deoxycholate, 0.1% SDS, 1 mM EDTA, 10% Glycerol) supplemented with protease (cOmplete, Roche) and phosphatase (Sigma–Aldrich) inhibitor cocktails on ice for 20 min. Homogenates were centrifuged at 2,000g for 10 min and proteins in the supernatants were denatured in Laemmli buffer at 95°C for 7 min. Otherwise, OPCs cultures were incubated with 2.5% perchloric acid followed by dialysis and dephosphorylation with alkaline phosphatase (Roche) as previously described (Iovino et al., 2015). Samples were resolved in 4–12% SDS‐PAGE gels (Invitrogen) and blotted on PVDF membrane (Merk). Membranes were blocked with 5% w/v skimmed milk dissolved in TBS containing 0.5% Tween (TBS‐T) and incubated overnight at 4°C with primary antibody diluted in TBST supplemented with 5% bovine serum albumin. Following incubation for 2 h at RT with HRP‐conjugated secondary antibody diluted (1:5,000) in 5% Milk TBST, membranes were visualized with ELC plus (GE). Recombinant human tau isoforms (tau ladder, Sigma–Aldrich) were used as positive control for the identification of the human transgene.

#### Elisa

One whole spinal cord was homogenized with a FastPrep‐24 homogenizer (MP Biomedicals) (2 × 10 s, 5,500 rpm) in a 5× volume of lysis buffer (50 mM Tris pH 7.4, 150 mM NaCl, and 5 mM EDTA) supplemented with protease and phosphatase inhibitor cocktails. Suspensions were centrifuged at 18,000*g* for 10 min, and supernatants used to detect cytokines with a multiplex ELISA plate (Multi Spot Assay system, Pro‐inflammatory Panel 1 kit, MSD, USA). Results were normalized for the protein concentration of each sample.

#### RT‐PCR

OPCs cultures were harvested with RTL lysis buffer, passed through the QIAshredder column (Qiagen), and the RNA extracted with the RNeasy columns (Qiagen, Crawley, UK). RNA was extracted from human brain in the same way. We loaded 500 ng of RNA of each sample for amplification with OneStep RT‐PCR (Qiagen). To detect human tau we amplified cDNA from exon 9 (forward primer: 5′ CTCCAAAATCAGGGGATCGC 3′) to exon 12 (reverse primer: 5′ TTTTTATTTCCTCCGCCAG 3′), at the following PCR conditions: 50°C for 30 min, 95°C for 15 min, 94°C for 30 s, 65°C for 30 s, 72°C for 1 min for 40 cycles. This primer pair produces separate bands from tau mRNAs with 3 (461 bp) or 4 repeats (552 bp) (Anfossi et al., 2011). mRNA from human brain was used as positive control. To detect mouse GAPDH as loading control we used the following primers: forward primer: 5′ TTCCAGTATGACTCTACCC 3′, and reverse primer: 5′ ATGGACTGTGGTCATGAGCCC 3′, at the following PCR conditions: 50°C for 30 min, 95°C for 15 min, 94°C for 30 s, 61°C for 30 s, 72°C for 1 min for 30 cycles.

### Imaging Analysis

All imaging analyses were carried out with ImageJ (1.49).

For the quantification either cell density or staining intensity we analyzed four to five slides for each animal. The lesion in lysolecithin‐injected spinal cords was identified as the area of white matter with substantially higher density of Hoechst‐positive nuclei. The contour of the lesion drawn in the blue channel was over‐imposed on the other channels before counting the cell number or measuring staining intensity.

### Statistical Analysis

Values are expressed as means ± SEM. Statistical analyses for significant differences were performed with unpaired *t* test for all the results except the ranking analysis data that were analyzed with Mann–Whitney test using GraphPad Prism 5.0 software (GraphPad Software, San Diego, CA). The criterion for statistical significance was *P* < 0.05.

## Results

### Early Signs of Axonal Pathology in the Spinal Cord of P301S‐htau Mice

**Figure 1:**
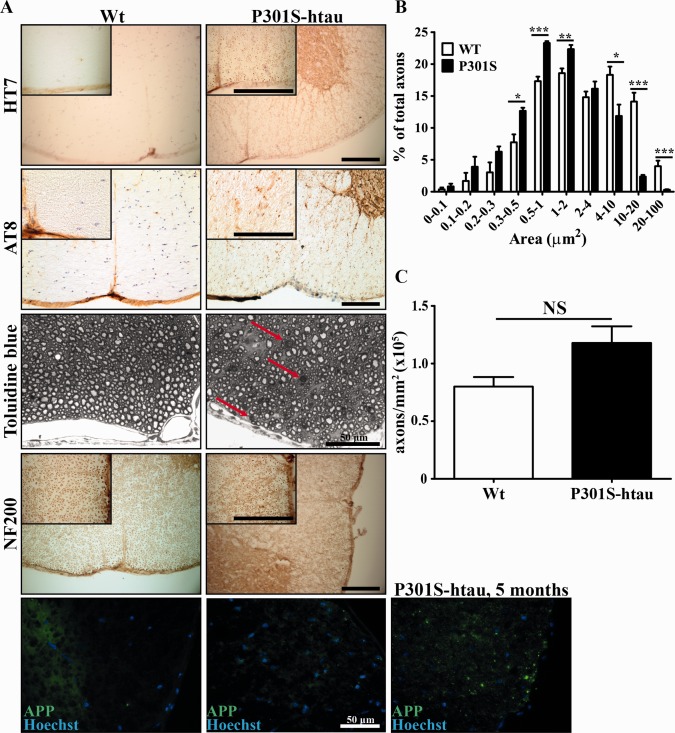
Early signs of axonal injury in the spinal cord of P301S‐htau mice. Thoracic (T12‐T13) spinal cord sections taken from 2‐month‐old Wt and P301S‐htau mice were stained with antibodies or toluidine blue (**A**). Red arrows in toluidine blue stained sections point to myelin figures in P301S‐htau mice. Myelin figures were absent in Wt mice. Quantification of axonal calibre distribution (**B**) and axonal density (**C**) in the ventral funiculus of 2‐month‐old Wt and P301S‐htau mice measured in toluidine blue stained sections. Scale bars represent 100 μm, if not otherwise indicated. Quantification of axonal calibre was obtained from three pictures of the ventral funiculus taken at ×100 magnification from four P301S‐htau and four Wt mice. Each picture contained 250–450 axons. Statistical analysis was performed between axons of Wt and P301S‐htau mice with the same calibre range using unpaired *t* test. NS = non‐significant, * = *P* ≤ 0.05, ** = *P* ≤ 0.01, *** = *P* ≤ 0.001. [Color figure can be viewed in the online issue, which is available at wileyonlinelibrary.com.]

The expression of the human tau transgene in P301S‐htau mice rapidly increases in the first 2 weeks of life to plateau at 4–5 weeks of age in association with the appearance of abnormal hyperphosphorylation and filamentous tau (Scattoni et al., 2010; Delobel et al., 2008). Consistently, in the ventral funiculus of 2‐month‐old P301S‐htau mice virtually every axon was immunoreactive for HT7 (phosphorylation‐independent human tau specific antibody recognizing the transgene), while several axons were also positive for AT8 immunostaining (phosphorylation‐dependent human and mouse tau antibody) (Fig. 1A). Despite the early development of tau pathology in P301S‐htau mice, significant neuronal loss has been detected only from 3 months of age (Hampton et al., 2010; Yang et al., 2015). Interestingly, in 2‐month‐old P301S‐htau mice toluidine blue staining of the ventral funiculus (embedded in resin) revealed a marked change in axonal calibre distribution and occasional myelin figures (concentrically layered myelin without axon) suggestive of Wallerian degeneration (Fig. 1, red arrows in C). Compared with Wt, in P301S‐htau mice the proportion of larger‐calibre axons (≥4 μm^2^) was significantly reduced while that of smaller‐calibre axons (≤2 μm^2^) was significantly increased (Fig. 1B). The total axonal density in P301S‐htau mice was not significantly different from Wt mice (Fig. 1C). Neurofilament staining with NF200 antibody showed that axons in the ventral funiculus of the spinal cord were consistently smaller and more condensed in P301S‐htau mice compared with Wt mice (Fig. 1D).

We next investigated whether the axons in the ventral funiculus of the spinal cord were immunoreactive for APP, which accumulates in degenerating axons (Ferguson et al., 1997; Sherriff et al., 1994). Two‐month‐old P301S‐htau mice displayed a limited number of APP positive axons that were mostly in the marginal areas, which are normally populated by larger‐calibre axons (Fig. 1A). Conversely, axonal degeneration was increased in 5‐month‐old P301S‐htau mice where several APP positive axons were present (Fig. 1A). These data suggest that in young adult P301S‐htau mice, axons exhibit signs of pathology without overt axonal degeneration.

### Early Microgliosis in P301S‐htau Mice

**Figure 2:**
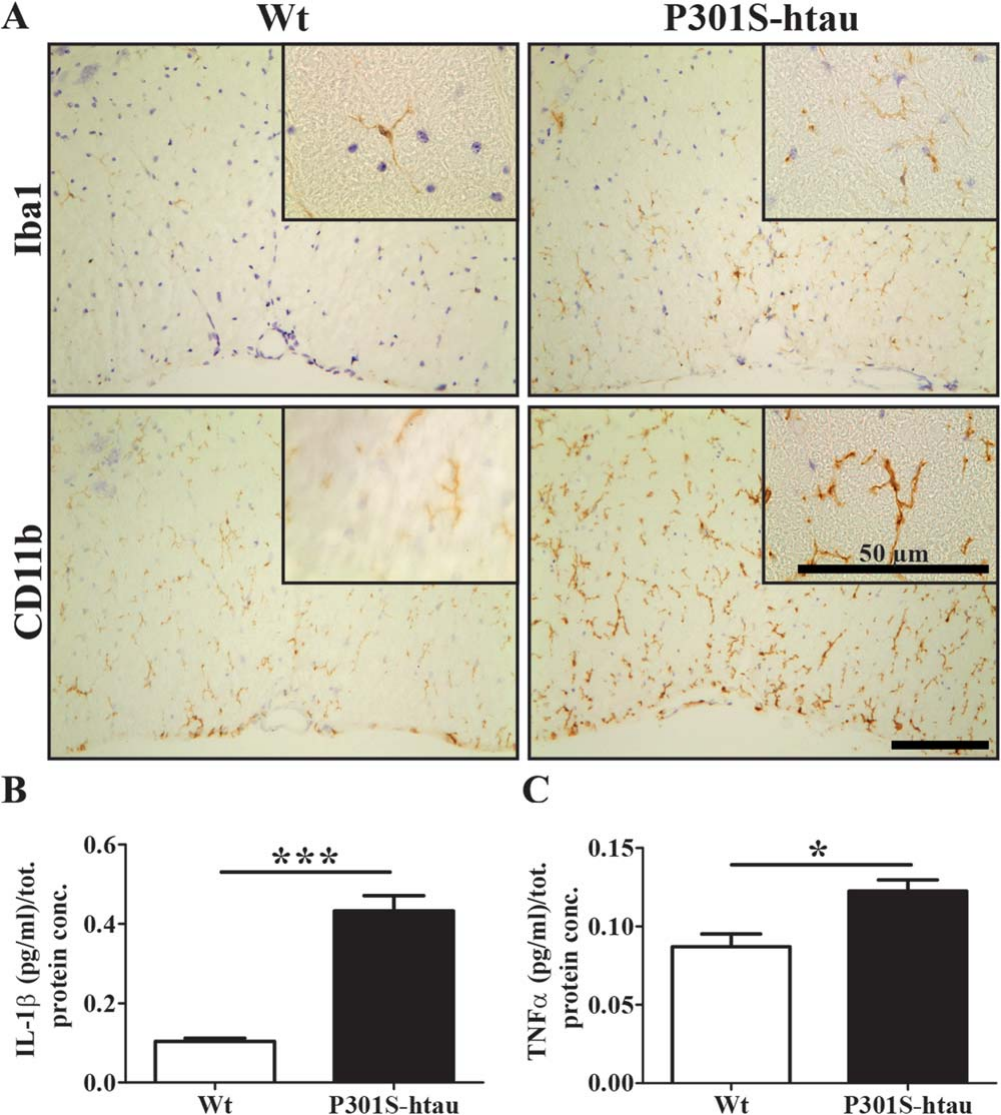
Early microgliosis in P301S‐htau mice. Thoracic (T12‐T13) spinal cord sections from 2‐month‐old Wt and P301S‐htau mice were immunostained for microglial markers (Iba1 and CD11b) (**A**). Scale bars represent 100 μm, if not otherwise indicated. Protein extracts from the whole spinal cord of 2‐month‐old Wt and P301S‐htau mice were assessed by ELISA for IL‐1β (**B**) and TNFα (**C**). Data represent the mean ± SEM from four P301S‐htau and four Wt mice. Statistical difference between Wt and P301S‐htau mice was calculated using unpaired *t* test. * = *P* ≤ 0.05, *** = *P* ≤ 0.001. [Color figure can be viewed in the online issue, which is available at wileyonlinelibrary.com.]

Two‐month‐old P301S‐htau mice showed a marked microgliosis in the spinal cord (Fig. 2A), as well as significantly increased levels of the inflammatory cytokines IL‐1β and TNFα (Fig. 2B,C), which was consistent with previously published work on older (5‐month‐old) P301S‐htau mice (Bellucci et al., 2004).

CD11b‐ and Iba1‐immunoreactive microglia/macrophages in 2‐month‐old P301S‐htau mice exhibited mostly ramified appearance with thick processes, despite occasional clustering and few amoeboid‐shaped cells. In contrast, most microglial cells from 5‐month‐old P301S‐htau mice were amoeboid with short processes and a propensity to cluster (Supporting Information Fig. 1). These data indicate an inflammatory response to early axonal pathology in P301S‐htau mice.

### Increased Density of Differentiated Oligodendrocytes After Focal Demyelination in P301S‐htau Mice

**Figure 3:**
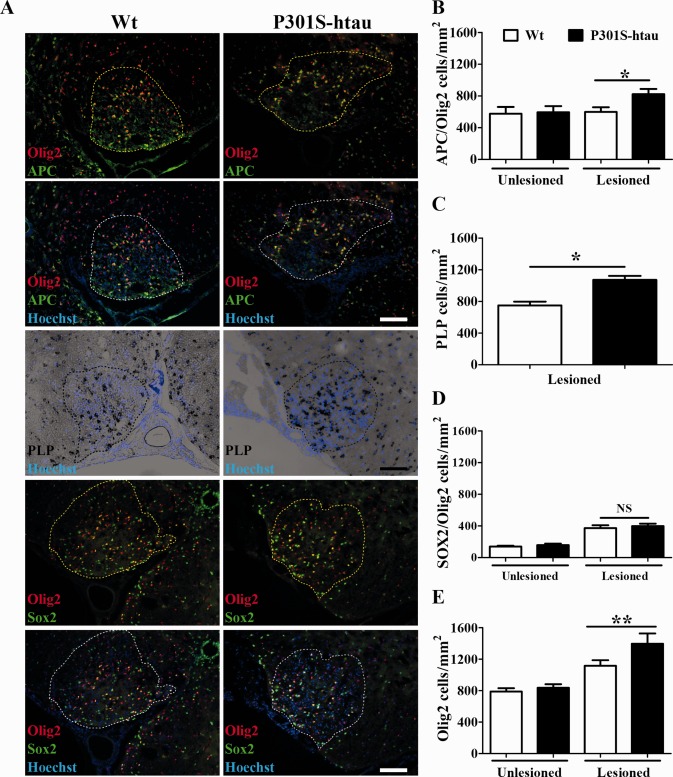
Increased density of differentiated oligodendrocytes after focal demyelination in P301S‐htau mice. Lysolecithin was injected in the ventral funiculus of thoracic (T12‐13) spinal cords of 2‐month‐old Wt and P301S‐htau mice. After 14 days from the injection tissues were collected, sectioned, and stained for oligodendrocyte (*in situ* hybridization for PLP, and immunofluorescence for APC) and OPCs markers (Olig2^+^/Sox2^+^ cells) (**A**). Scale bars: 100 μm. Quantification of APC^+^/Olig2^+^ cells within and outside the lesioned white matter (**B**). Quantification of PLP mRNA^+^ cells within the lesioned white matter (**C**). Quantification of Sox2^+^/Olig2^+^ cells within and outside lesioned white matter (**D**). Quantification of total number of Olig2^+^ cells within and outside the lesioned white matter (**E**). Data represent the mean ± SEM from at least six animals per group. Statistical analysis of cell number differences within the lesioned areas were calculated between Wt and P301S‐htau mice using unpaired *t* test. * = *P* ≤ 0.05, ** = *P* ≤ 0.01. [Color figure can be viewed in the online issue, which is available at wileyonlinelibrary.com.]

We next investigated the response to demyelination in P301S‐htau mice. Following a lysolecithin injection into the ventral funiculus of young adult mice, which induces a focal area of primary demyelination, OPCs migrate and proliferate around and within the lesion for the first few days. OPCs then start to differentiate into new myelin sheath forming oligodendrocytes at around 10 days post lesion (dpl)‐induction, such that at 14 dpl remyelination is well underway and usually complete at 21 dpl (Zhao et al., 2005). We quantified the number of OPCs and oligodendrocytes within the lesion (identified as the area of white matter with substantially higher density of Hoechst‐positive nuclei). Adenomatous polyposis coli (APC) protein immunostaining and proteolipid protein (PLP) *in situ* hybridization, markers of mature oligodendrocytes, demonstrated that 14 dpl P301S‐htau mice compared with Wt mice exhibited significantly higher density of oligodendrocytes within the lesion (Fig. 3A,B). Whereas the number of OPCs, identified as Sox2 (highly expressed in reactive OPCs) and Olig2 (an oligodendroglial linage marker) double positive cells, was the same within lesions in Wt and P301S‐htau mice (Fig. 3A,D). Mutant and Wt mice had the same density of mature oligodendrocytes (Olig2^+^/APC^+^ cells) and OPCs (Sox2^+^/Olig2^+^) in unlesioned white matter (Fig. 3B,D). Quantification was inclusive of most of the oligodendroglial lineage, since the sum of APC^+^/Olig2^+^ and Sox2^+^/Olig2^+^ cells closely mirrored the total number of Olig2^+^ cells (Fig. 1B,D,E). Because of a reduction of axonal calibre average in naïve P301S‐htau mice compared with Wt mice, we investigated whether the lesion size in the two mouse lines differed. The lesion areas were identical between mutant and Wt mice (Supporting Information Fig. 2), suggesting that the changes in oligodendrocytes density was not due to shrinkage of the lesion size in P301S‐htau mice.

### Remyelination and Axonal Degeneration

**Figure 4:**
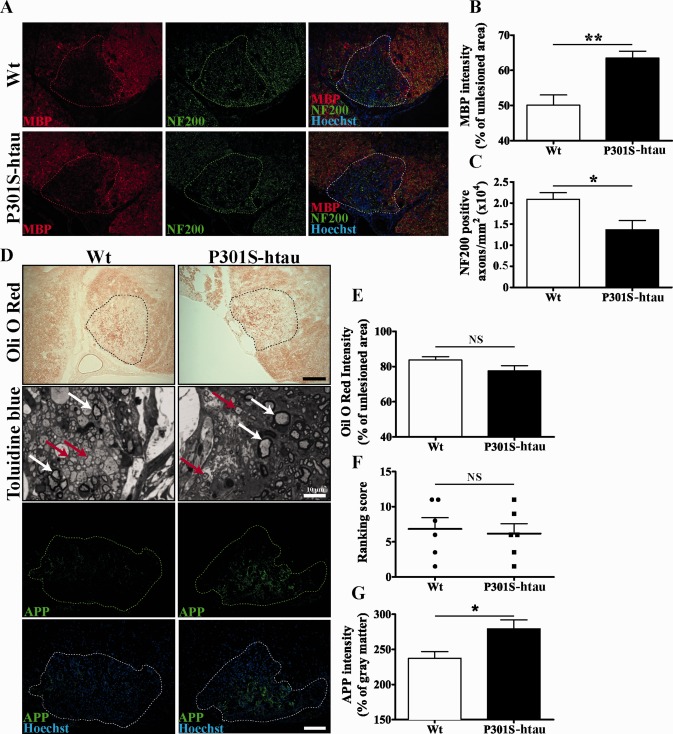
Remyelination and axonal degeneration. Lesioned spinal cords from 2‐month‐old Wt and P301S‐htau mice 14 days after lysolecithin injection were sectioned and stained (**A** and **D**). In the toluidine blue stained images, red arrows point to examples of remyelinated axons, whereas white arrows point to axons that had not been demyelinated. Scale bars represent 100 μm, if not otherwise indicated. Quantification of MBP intensity within the lesioned area is calculated as % of non‐lesioned white matter within the same section (**B**). Quantification of NF200 positive axons within the lesioned area (**C**). Quantification of Oli O Red staining intensity (marker of myelin debris) within the lesioned area is calculated as % of non‐lesioned white matter within the same section (**E**). Ranking analysis (see Methods) of toluidine blue stained sections (**F**). Quantification of APP staining intensity within the lesioned area represented as % of grey matter within the same section (**G**). Data represent the mean ± SEM from at least six animals per group. Statistical analysis was performed between Wt and P301S‐htau mice using Mann–Whitney test for the ranking score (F) and unpaired *t* test for the other data (B, C, E, and G). * = *P* ≤ 0.05, ** = *P* ≤ 0.01. NS = non‐significant. [Color figure can be viewed in the online issue, which is available at wileyonlinelibrary.com.]

We next investigated the degree of remyelination. Figure 4A shows that at 14 dpl myelin basic protein (MBP) was significantly more abundant within the lesion of P301S‐htau than Wt mice (Fig. 4A,B). As MBP immunostaining fails to distinguish myelin sheaths from myelin debris, we stained the sections with Oil O‐Red, which within the lesion strongly highlights lipid droplets representing myelin debris. Both mutant and Wt mice presented similar degrees of myelin debris within the lesions (Fig. 4D,E), suggesting that the presence of higher levels of MBP in P301S‐htau mice was due to an increase of new myelin sheaths. To investigate further the degree of remyelination we analyzed the presence of remyelinated axons in semi‐thin sections of resin embedded tissue. The myelin thickness visualized by toluidine blue staining allows differentiation of axons that have been remyelinated (thin myelin sheath, red arrows) from normally myelinated axons that have not been demyelinated by lysolecithin (thick myelin sheath, white arrows) (Blakemore, 1974). Ranking analysis of the extent of remyelination did not reveal a significant difference between transgenic and control mice (Fig. 4D,F). We did, however, find a marked reduction of axons within the lesion of P301S‐htau compared with Wt mice, which represented a 35% reduction in NF200 positive axons (Fig. 4A,C). We also observed a greater amount of APP immunoreactivity in the lesion in P301S‐htau compared with Wt mice (Fig. 4D,G). However, in non‐lesioned spinal cords of P301S‐htau mice there was only a limited number of APP positive axons and the axonal density in the ventral funiculus was not significantly different between transgenic and Wt mice (Fig. 1A,C).

These data demonstrate that following demyelination P301S‐htau mice develop within the lesion mature oligodendrocytes that are able to produce more myelin sheaths compared with Wt mice. However, the reduced number of axons available for myelination in the lesion in P301S‐htau compared with Wt mice renders it difficult to unequivocally determine differences in the remyelination capacity between transgenic and Wt mice.

### OPCs Isolated from P301S‐htau Mice Showed Enhanced Differentiation Capacity

**Figure 5:**
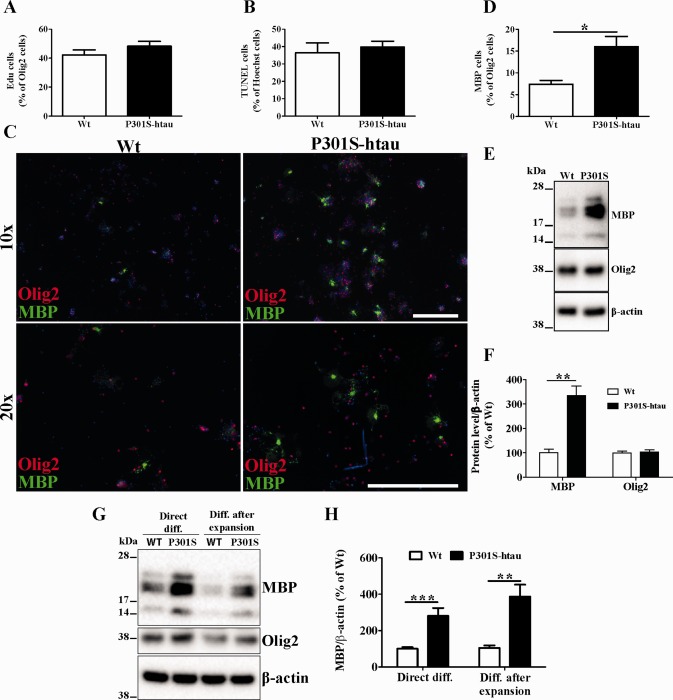
OPCs isolated from P301S‐htau mice showed enhanced differentiation. OPCs were isolated from P10‐12 Wt and P301S‐htau mice, and grown in medium with or without growth factors (see Methods). Quantification of OPCs proliferation was performed 2 days after isolation in the presence of growth factors by counting the number of cells that incorporated EdU (**A**). Quantification of cell death 4 days after growth factors withdrawal was performed by TUNEL staining (**B**). OPCs cultures were immunostained for MBP and Olig2 4 days after growth factors withdrawal (**C**) and MBP^+^ cells were represented as % of Olig2^+^ cells (**D**). Protein extracts from OPCs cultured after 4 days of growth factors withdrawal were probed for MBP, Olig2, and β‐actin (**E**) and bands intensity was represented as % of Wt cultures (**F**). OPCs isolated from P10‐12 Wt and P301S‐htau mice were grown in the presence of growth factors for either 4 (direct diff.) or 10 days (diff. after expansion) before proteins were extracted to detect the MBP levels (G). Data represent the mean ± SEM from at least 4 (A, B, D) or 3 (F and H) independent experiments. Statistical analysis performed between Wt and P301S‐htau mice using unpaired *t* test. * = *P* ≤ 0.05, ** = *P* ≤ 0.01, *** = *P* ≤ 0.001. [Color figure can be viewed in the online issue, which is available at wileyonlinelibrary.com.]

The increased axonal degeneration observed in P301S‐htau mice after demyelination may have influenced the rate of OPC differentiation. Thus, we compared the properties of OPCs derived from mutant and Wt mice in an *in vitro* system where axons are absent. OPCs from mutant and Wt mice comparably migrated towards a higher concentration of growth factors (data not shown) and proliferated as measurement of the DNA integration of EdU over a 3‐h period (Fig. 5A). In the presence of growth factors‐free medium most OPCs either differentiate in oligodendrocytes or die by apoptosis (Miron et al., 2013). Following withdrawal of growth factors for 4 days, we found a similar number of TUNEL^+^ cells but a twofold increase of MBP^+^/Olig2^+^ mature oligodendrocytes in cultures derived from P301S‐htau mice compared with those from Wt mice (Fig. 5B–D). This was corroborated by a threefold increase of MBP protein in extracts obtained from cultures derived from P301S‐htau mice compared with Wt mice (Fig. 5E,F).

Next we asked how profoundly OPCs isolated from P301S‐htau have been influenced by the presence of pathological tau *in vivo*. To this end, isolated OPCs from 10‐day‐old mice were cultured with growth factors for either 4 (direct differentiation) or 10 (differentiation after expansion) days before inducing differentiation. To ensure the presence of a comparable population between Wt and P301S‐htau‐derived OPCs after the *in vitro* cell expansion, we sorted for a second time cells with A2B5‐conjugated microbeads (see Methods). Figures 5G,H show that OPCs isolated from P301S‐htau mice retained the capacity to produce more MBP compared with those derived from Wt mice during differentiation after a prolonged cell expansion.

These results demonstrated that OPCs from P301S‐htau mice differentiate into mature oligodendrocytes more efficiently than those from Wt mice even in culture in the absence of axonal degeneration. Also OPCs isolated from P301S‐htau mice retained this enhanced differentiation capacity even after a prolonged cells expansion.

### Human Tau Protein is not Ectopically Expressed or Found in P301S‐htau OPCs

**Figure 6:**
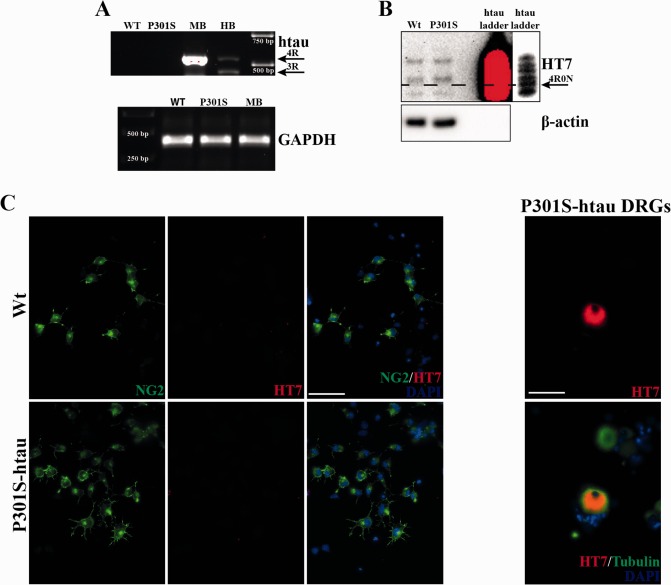
Human tau protein is not ectopically expressed or found in P301S‐htau OPCs. RNA extracted from OPC cultures 48 h after isolation were probed for the presence of htau mRNA (**A**). Both Wt and P301S‐htau OPCs showed no presence of htau. RNA extracts of P301S‐htau mouse (MB) and human brain (HB) were used as positive control and show a strong 4R band, and 4R and 3R band tau respectively. Mouse GAPDH used as control shows that the amount of mRNA used was similar in all mouse samples (A). Protein extracts of OPCs cultures for 48 h after isolation were dephosphorylated with alkaline phosphatase and probed for HT7 (anti‐human specific phosphorylation‐independent antibody) (**B**). No specific bands were stained in protein extracts of both Wt and P301S‐htau OPCs culture. Recombinant human tau ladder was used as marker to identify the human tau bands using a lower exposure time compared with the cell extracts (ladder on the right). OPCs cultures for 48 h after isolation were immunostained with NG2 (plasma membrane marker of OPCs) and HT7 (**C**). No HT7 immunoreactivity was observed in NG2^+^ cells. Dorsal root ganglion (DRG) culture obtained from 2‐month‐old P301S‐htau mice was used as positive control of HT7 immunostaining in βIII‐tubulin (Tubulin) neurons. Scale bars represent 50 μM. [Color figure can be viewed in the online issue, which is available at wileyonlinelibrary.com.]

The expression of P301S‐htau transgene is restricted to the nervous system by the use of Thy1.2 promoter that contains only the neuronal‐specific element of the Thy1 promoter (Caroni, 1997; Vidal et al., 1990). However, to confirm that the transgene was not ectopically expressed in OPCs, we measured the expression of human tau in OPCs isolated from P301S‐htau mice and compared it to the total expression of P301S‐htau mice and human brain tissues (Fig. 6A). RT‐PCR for 3‐repeat (3R) and 4‐repeat (4R) tau showed the presence of two bands in human brain (HB), one band corresponding to the transgene in P301S‐htau mouse brain (MB), but no band was observed in OPC extracts.

Compelling evidence demonstrates that an increasing variety of cell types release and uptake pathological tau (Clavaguera et al., 2015). Thus, we investigated the presence of human tau protein in isolated OPCs. Protein extracts from freshly isolated OPCs from P301S‐htau mice showed no specific band when probed with HT7 antibody even after a prolonged exposure (Fig. 6B). Also OPCs expressing NG2 (a proteoglycan expressed on the plasma membrane of OPCs) that were kept proliferating 48 h after isolation from P301S‐htau mice showed no immunoreactivity for HT7, which in contrast was readily detected in dorsal root ganglion culture derived from P301S‐htau mice (Fig. 6C).

Even though OPCs from P301S‐htau mice contained no human tau, their functionality may be influenced by exposure to the transgenic protein *in vivo*, which has been shown to be released by tau‐overexpressing neurons (de Calignon et al., 2012). To test this hypothesis, we exposed OPCs isolated from Wt mice to filamentous and hyperphosphorylated tau obtained by sarkosyl extraction of spinal cords of 5‐month‐old P301S‐htau mice as previously described (Goedert and Jakes, 1990). A range of concentrations of sarkosyl‐insoluble extracts (1.5, 7.5, 15 μg mL^−1^) incubated for 4 days during the growth factor withdrawal phase failed to enhance the differentiation of Wt mice‐derived OPCs (data not shown). These data suggest that the increased differentiation capacity of isolated P301S‐htau OPCs is not dependent on the human transgene expression or spreading of tau to these cells *in vivo*.

### The Enhanced Differentiation Capacity of P301S‐htau Mice‐derived OPCs is Acquired

**Figure 7:**
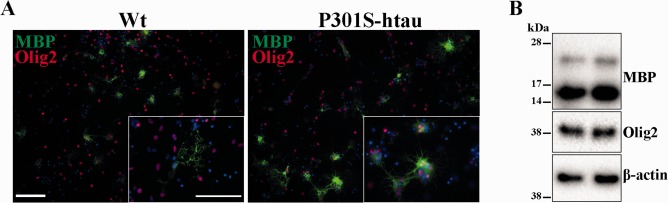
The enhanced differentiation capacity of P301S‐htau OPCs is acquired. OPCs isolated from postnatal day 1 Wt and P301S‐htau mice were subjected to the same differentiation protocol as described (**A** and **B**). Following 4 days of growth factors withdrawal cells were either fixed and immunostained for MBP and Olig2 (A) or lysed for protein extraction (B). Scale bars represent 100 μM. [Color figure can be viewed in the online issue, which is available at wileyonlinelibrary.com.]

We previously showed that in the brain of P301S‐htau mice the human tau protein is present at low level at birth and rapidly increases up to 14 days of age (Scattoni et al., 2010). Thus, to assess the relationship between the level of transgene expression and OPC differentiation rate, we repeated the protocol on OPCs isolated 1 day after birth. In this new condition OPCs derived from P301S‐htau mice differentiated into MBP positive cells bearing myelin sheath to a similar degree as Wt mice‐derived OPCs (Fig. 7A). Also MBP protein level in extracts from cultures of OPCs isolated from either P301S‐htau or Wt mice following growth factor withdrawal were almost identical (Fig. 7B). The expression pattern of MBP isoforms in the cultures derived from newborn mice was different from those derived from older mice. Changes in the level of MBP isoform expression during the different stage of development have previously been described (Mathisen et al., 1993).

## Discussion

This work shows for the first time that axonal injury, due to the expression of pathological tau, promotes the differentiation of OPCs during remyelination.

Most axons in the ventral funiculus of the spinal cord in P301S‐htau mice expressed the human transgene and several exhibited hyperphosphorylated tau at 2 months of age. At this age P301S‐htau mice displayed atrophic axons in the absence of a marked axonal degeneration, suggesting an early injury state (Fig. 1). This is in line with the axonal transport impairment, both anterograde and retrograde, that we recently reported *in vivo* in the optic nerve of the P301S‐htau mice, *in vitro* in dorsal root ganglion neuronal cultures and human IPS‐derived neurons (Bull et al., 2012; Iovino et al., 2015; Mellone et al., 2013). Similar results were observed also by Ittner et al. (2008) in transgenic mice expressing human tau with the K369I mutation. Axonal transport impairment is also present in MS lesions (Ferguson et al., 1997), which may be linked to the presence of tau hyperphosphorylation that we have previously described (Anderson et al., 2008, 2009, 2010). In P301S‐htau mice the early axonal injury was associated with microgliosis creating an inflammatory environment (Fig. 2). By 5 months of age P301S‐htau mice showed activated microglial cells that were mostly amoeboid‐shaped and clustered (Supporting Information Fig. 1) (Bellucci et al., 2004). Similar microglial clustering is frequently found in early stages of MS white matter in the absence of demyelination, but associated with axonal injury identified as accumulation of APP and non‐phosphorylated neurofilament (Singh et al., 2013). To note, an inflammatory microenvironment is essential for an efficient remyelination, for example for the removal of myelin debris from the demyelinated lesions (Kotter et al., 2005; Miron et al., 2013; Ruckh et al., 2012). Importantly, the cytokines IL‐1β and TNFα, which are upregulated in young P301S‐htau mice compared with Wt mice (Fig. 2), are instrumental to the differentiation of OPCs and remyelination after a toxin‐induced demyelination (Arnett et al., 2001, 2003; Mason et al., 2001; Zhao et al., 2006). Therefore, the inflammatory environment present in P301S‐htau mice before lysolecithin injection may be responsible for the increased density of mature oligodendrocytes (Fig. 3). This increase of oligodendrocytes in P301S‐htau mice compared with Wt mice may have resulted from either an enhanced recruitment of OPCs within the first few days after the lesion or from a more efficient OPC differentiation. Despite the fact that enhanced OPC differentiation was specifically associated with an increase of myelin sheath, we were not able rigorously to compare the degree of remyelination between transgenic and Wt mice due to an increased axonal degeneration in P301S‐htau mice following lysolecithin injection (Fig. 4). In fact although lysolecithin normally causes only limited axonal degeneration (Huang et al., 2012), lesions in P301S‐htau mice showed a 35% reduction of NF200 positive axons and a significant increase of APP accumulation compared with Wt mice (Fig. 4). This may be due to lysolecithin‐induced depletion of myelin‐derived trophic support to axons, which in the case of already damaged P301S‐htau axons resulted in marked degeneration. Indeed Nikic et al.(2011) have described that in an experimental model of MS axons showing signs of injury such as swelling and dystrophic mitochondria, can recover if they retain the myelin wrapping. To determine whether the axonal degeneration observed in P301S‐htau mice may have influenced the OPCs maturation *in vivo*, we investigated the functionality of OPCs isolated from 10‐ to 12‐day old mice. Interestingly, we found that OPCs from P301S‐htau mice more efficiently differentiated into mature oligodendrocytes with formation of membrane sheets (Fig. 5D–F). Further we demonstrated that this enhanced differentiation capacity of OPCs derived from P301S‐htau mice is maintained after a 10‐day cell expansion, representing about four cell replications (Fig. 5G,H). This supports the *in vivo* data showing that the increased OPC differentiation in transgenic compared with Wt mice did not require the presence of axonal degeneration.

We also excluded the possibility that the phenotype found in P301S‐htau mice‐derived OPCs was due ectopic expression or spreading of the human transgene into these cells (Fig. 6). Thus we reasoned that OPCs from P301S‐htau mice may be primed *in vivo* by a microenvironment that reacts to neuronal expression of the transgene. This hypothesis was further supported by absence of the enhanced differentiation capacity of OPCs isolated from 1‐day‐old P301S‐htau mice when the level of transgene expression is limited (Fig. 7A,B). Therefore the level of transgenic protein and the time that OPCs are exposed to the P301S‐htau mice *in vivo* microenvironment are essential for their priming.

We speculate that the hyperphosphorylation of tau induced by inflammatory attacks of the CNS in MS contributes to early axonal injury, which in turn may signal to OPCs to facilitate remyelination. In case remyelination fails, the hyperphosphorylation of tau may contribute to toxic oligomerization and fibril formation leading to the degeneration of axons driving the clinical symptoms of progressive MS where indeed hyperphosphorylated and aggregated tau is present (Anderson et al., 2008). Future studies will investigate the specific molecules involved in the communication between axons and OPCs that leads to an improved maturation of oligodendrocytes in our model of tauopathy.

## Supporting information

Supporting Information

Supporting Information

Supporting Information
