# Supplementary figures and images for: Neuronal expression of pathological tau accelerates oligodendrocyte progenitor cell differentiation
Source: Glia. 2015 Nov 18;64(3):457–71. doi: 10.1002/glia.22940 (PMC5132073; doi:10.1002/glia.22940)

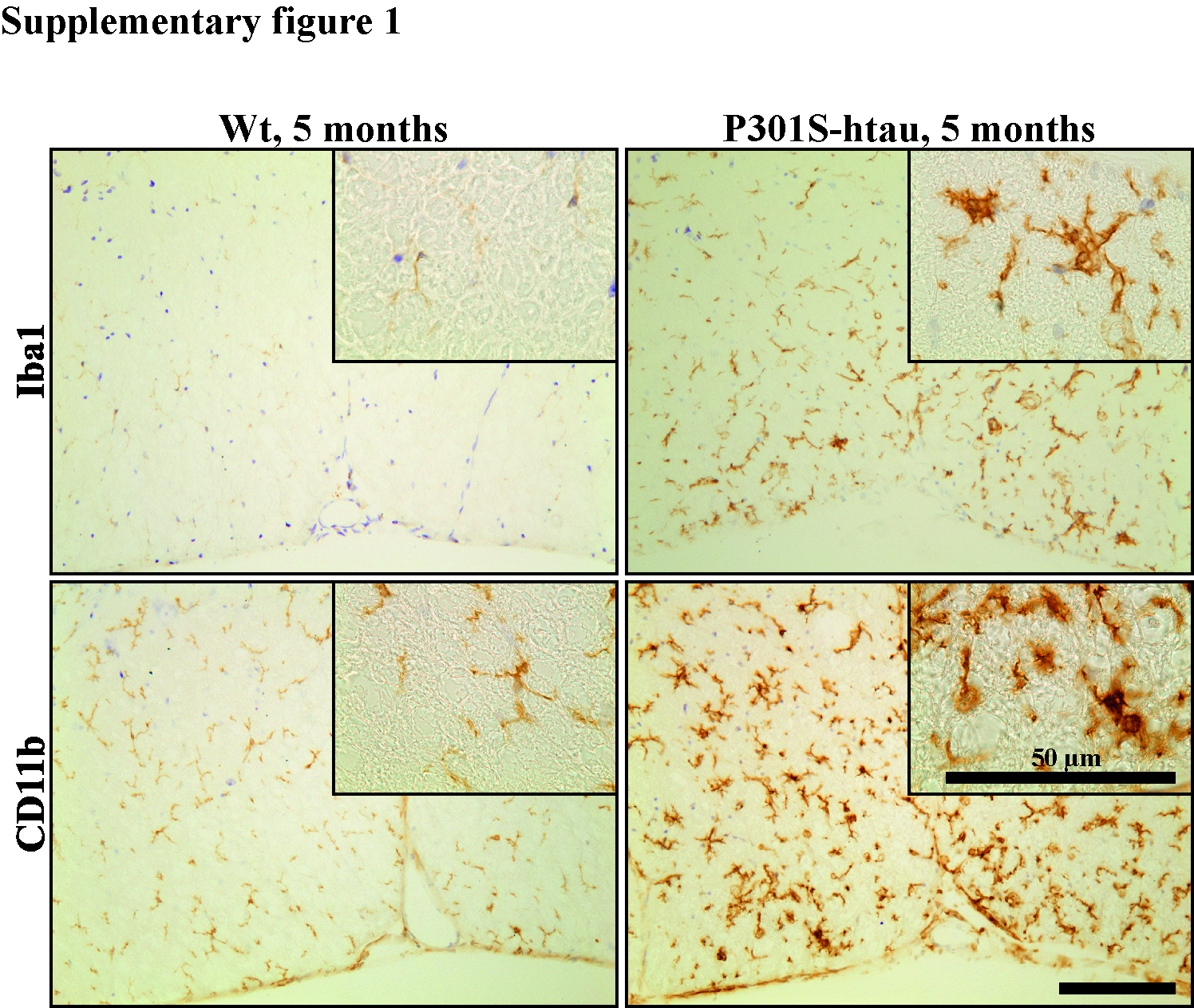

Supplement: Supplementary file 1 — Supporting Information [file GLIA-64-457-s001.tif]

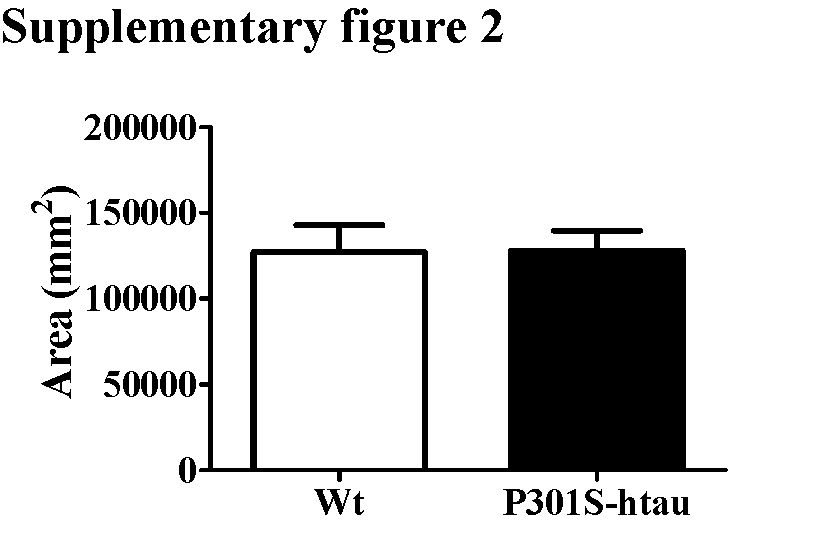

Supplement: Supplementary file 2 — Supporting Information [file GLIA-64-457-s002.tif]
