# Supplementary material for: Neuronal expression of pathological tau accelerates oligodendrocyte progenitor cell differentiation
Source: Glia. 2015 Nov 18;64(3):457–71. doi: 10.1002/glia.22940 (PMC5132073; doi:10.1002/glia.22940)
Supplement: Supplementary file 3 — Supporting Information [file GLIA-64-457-s003.doc]

**Neuronal expression of pathological tau accelerates oligodendrocyte progenitor cell differentiation**

Bernardino Ossola^1^, Chao Zhao^1,2^, Alastair Compston^1^, Stefano Pluchino^1^,

Robin J.M. Franklin^1,2^, Maria Grazia Spillantini^1^*****

^1^ Department of Clinical Neurosciences, University of Cambridge, Clifford Allbutt Building, Cambridge CB2 0QH, UK

^2^ Wellcome Trust-Medical Research Council Cambridge Stem Cell Institute, University of Cambridge, Clifford Allbutt Building, Cambridge CB2 0QH, UK

**Supplementary methods**

**OPCs cultures**

Cortical hemispheres were finely chopped with springbow dissecting scissors and digested for 30 min at 37°C with papain (20 U/ml, Worthington, UK). After adding ovomucoid inhibitor (Worthington, UK), the tissue was gently triturated with fire-polished glass pipettes and subsequently passed through a 70 μm cell strainer. Only for tissue derived from 10-12-day-old mice myelin debris was removed by centrifuging 3.5 mL of 90% isotonic Percoll (GE, Sweden) added to 11 mL of cell suspension for 20 min at 800 g. The myelin debris layer was removed and the pellet was washed once with HBSS solution (Invitrogen, UK). OPCs were incubated with A2B5-conjugated microbeads (Miltenyi Biotec, San Francesco, CA, USA) and isolated for positive selection with appropriate sized columns. OPCs were resuspended in OPCs medium (DMEM supplemented with 1% penicillin/streptomycin, 1% N2 supplement (Invitrogen), 2% B27 supplement (Invitrogen), 0.1% N-Acetyl-L-cysteine (Sigma-Aldrich), 0.1% biotin (Sigma-Aldrich), 0.1% Trace Element B (Cellgro), and 0.1% Forskolin (Sigma-Aldrich) and seeded into multi-well plates coated with poly-D-lysine at a density of 35000 cells/cm^2^. Cells were grown at 37°C with 5% CO_2_ in the presence of growth factors (10 ng/ml ciliary neurotrophic factor (CNTF, Proteintech, USA), 20 ng/ml PDGFAA (Proteintech), 1 ng/ml neurotrophin 3 (NT3, Proteintech)) and half of the medium was changed every two days maintaining the same final concentration of growth factors.

For the cell expansion experiment OPCs were grown in T75 flasks for 10 days in the presence of growth factors. After detaching with papain (2 U/ml) cells were sorted with A2B5-conjugated microbeads (see above) to ensure the use of undifferentiated OPCs. Cells were plated into multi-well plates coated with poly-D-lysine at a density of 35000 cells/cm^2^ in the presence of growth factor-free OPC medium for four days.

**Immunohistochemistry and immunocytochemistry**

Mice were terminally anesthetized with sodium pentobarbital (Euthatal; 2 ml/kg; Rhône-Mérieux) and transcardially perfused with 50 ml PBS followed by 50 ml of 4% paraformaldehyde (PFA) (Merck) dissolved in PBS pH 7.4. Spinal cords were post-fixed in 4% PFA overnight at 4°C, washed and then cryoprotected in PBS containing 25% sucrose and 0.02% sodium azide at 4°C for at least 48h. Tissues were then embedded in OCT mounting medium (Thermo-Fisher) and stored at -80°C. Sections of the spinal cord (12 or 20 μm) were mounted onto positively charged glass slides (SuperFrost Plus; VWR) and sorted at -20°C. For colorimetric immonostaining the tissue’s peroxidase activity was quenched with 3% H_2_O_2_ in PBS containing 20% MetOH for 30 min at RT. Sections were then blocked with PBS/0.3% Triton-X 100 (PBST) containing 5% serum of appropriate species for 2h at RT, and incubated overnight at 4°C with primary antibodies diluted in PBST in the presence of 5% serum for non-tau antibodies. For fluorescence staining sections were incubated with the appropriate secondary antibody conjugated with Alexa fluorochromes (Life Technology) and nucleus dye Hoechst 33258 or DAPI (1 μg/ml) diluted in PBST for 2h at RT. Slides were mounted with FluorSafe (MerckMillipore) and fluorescence was visualized using a LeicaTMDM6000B fluorescence microscope and images were acquired with Leica LAS AF software. For colorimetric detection sections were incubated with the appropriate biotin-conjugated secondary antibody (1:1000 in PBST) for 2h at RT followed by incubation with ABC kit (Vector) for 1h at RT. Staining was developed with 3,3 diaminobenzidine (Vector) and imaged with Olympus BX50 upright microscope.

**Supplementary figure 1. Late microgliosis in 5 month-old P301S-htau mice**. Thoracic (T12-T13) spinal cord sections (20 μm) from 5 month-old Wt and P301S-htau mice were immunostained for microglial markers (Iba1 and CD11b). Scale bars represent 100 μm, if not otherwise labelled.

**Supplementary figure 2. Comparison of P301S-htau and Wt mice lesion size.** From the images used to quantify OPC and mature oligodendrocyte density, we calculated the average lesion area (see methods) of lysolecithin-injected P301S-htau and Wt mice. Data represent the mean ± SEM from eleven P301S-htau and ten Wt mice. Statistical difference between Wt and P301S-htau mice was calculated using unpaired t-test.
